# Editorial: Understanding Urban Health Disparities: Family Planning Access and Use Among the Urban Poor in Low- and Middle-Income Countries

**DOI:** 10.3389/fgwh.2022.855435

**Published:** 2022-06-22

**Authors:** James Duminy, Onikepe Oluwadamilola Owolabi, Moazzam Ali, Moses Tetui

**Affiliations:** ^1^School of Geographical Sciences, University of Bristol, Bristol, United Kingdom; ^2^African Centre for Cities, University of Cape Town, Cape Town, South Africa; ^3^Vital Strategies, New York, NY, United States; ^4^World Health Organization, Geneva, Switzerland; ^5^School of Pharmacy, University of Waterloo, Waterloo, ON, Canada

**Keywords:** family planning, urban poverty, urban inequalities, LMICs, urbanization

With a growing majority of the world's population living in towns and cities, the future of the planet is increasingly urban. Indeed, urban processes in low- and middle-income countries (LMICs) will shape the future of global health and sustainability. For every 10 people added to the global urban population by 2050, nine will live in towns or cities of sub-Saharan Africa and Asia (1). Much of this growth will take place in conditions of material deprivation and weak public governance, often within the slums and informal settlements that dominate the public and policy imagination (2). Globally, around one billion people already live in slum-like conditions (3).

Recognizing the scale of this urban challenge, the Sustainable Development Goals (SDGs) include targets (under SDG 11: the “urban goal”) to promote more sustainable and inclusive urbanization and urban growth, involving a reduction in the proportion of urban residents living in slums and informal settlements. However, unless the urbanization of poverty—the trend for poverty to be concentrated in urban rather than rural areas—and the deepening of urban inequalities in LMICs are urgently addressed, both global and national targets for health and wellbeing will be derailed (4).

A starting point for this Research Topic is the conviction that improved access to safe and effective family planning (FP) services can make a major contribution to promoting health and wellbeing, economic development, gender equality, and environmental sustainability in towns and cities of LMICs, and in populations more generally. However, we currently lack adequate and detailed knowledge on the dynamics of FP access and use in urban areas of LMICs. This Research Topic contributes to this growing body of knowledge. All the articles address critical knowledge gaps by examining spatial inequalities in FP access and use, or by conducting studies of vulnerable urban groups.

## Spatial Inequalities in FP Access and Use

The articles included in this issue include several studies of geographic inequalities in FP supply, access and use, in some cases examining the links between geographic and economic inequalities. The authors investigate these distributions and inequalities at a range of scales. The study by Ross, for example, uses Demographic and Health Survey (DHS) data to compare urban and rural patterns of contraceptive use, access to methods, and fertility in six geographic regions. Providing the broader context for more detailed studies of urban contraception and fertility change, Ross helpfully points to the difficulties of making firm conclusions about either “urban advantage” or “urban penalty” when rural/urban dynamics and discrepancies vary so significantly between regions and, indeed, individual countries.

Other articles consider the relationship between FP use and the economic characteristics of urban populations across a range of countries. In their study, Akinyemi et al. find consistent associations between higher levels of women's deprivation and lower rates of contraceptive prevalence in five countries of West Africa—although that picture is far less consistent with respect to long-acting reversible methods in particular. Meanwhile, Hellwig et al. uncover significant differences in the links between economic inequality and the satisfaction of FP demand among a wide range of African countries. Both studies reveal that poorer urban women tend to enjoy lower levels of FP access and use than wealthier groups, but also that the nature of this trend can differ significantly between national contexts, both overall and in relation to specific FP methods.

Several articles offer a more localized and granular understanding of FP access and use by analyzing intra-urban distributions and differences. Tetui et al. analyze urban geographic distributions of FP supply *via* a survey of healthcare facilities in informal settlements of Kira Municipality, north-east of Kampala (Uganda). There, as in many other African settings (see the review by Duminy et al. in this Research Topic), poor urban residents tend to rely on private facilities that provide relatively low levels of access to long-acting contraceptive methods. In the same area, Lukyamuzi et al. focus on differences in the FP quality of care offered in formal and informal settlements, showing that informal residents tend to experience a lower quality of service, and express less satisfaction with that service, than those living in formally-developed areas.

## Studies of Vulnerable Groups

With most FP research in LMICs focusing on large urban centers (see the paper by Duminy et al.), we urgently require more knowledge on FP supply and demand in rapidly changing urban contexts, including secondary or medium-sized cities and peri-urban areas.

Three articles in this Research Topic provide insights into dynamics of a particular peri-urban site in the outskirts of Kampala (Uganda). Using a cross-sectional survey design, Birabwa et al. investigate knowledge and information exposure among women living in informal settlements, and conclude that high levels of awareness are not directly associated with rates of FP use. Tetui, Baroudi et al. examine FP demand, use and unmet need in the same area, demonstrating that local levels of unmet need are far higher than those presented by national urban estimates. Using focus group data, Mulubwa et al. focus on understanding the motivations to use contraceptives among young adults and adolescents in informal settlements, noting that motivations vary according to sources of information about contraceptives as well as social norms that deem contraceptive use unacceptable for unmarried adolescents.

In a different vein, Bose et al. offer their reflections on initiatives introduced by the Challenge Initiative in South Asia and Sub-Saharan Africa to improve access to contraception among young people living in urban slums. This was done by offering coaching programmes and the creation of an online learning platform to support city governments. Their work links to ongoing programmatic and research efforts to improve the organization of supply systems to improve FP supply in urban areas (5–7). The paper highlights that capacity building to secure trained and competent personnel to make and implement decisions is a critical precondition for improving the accessibility and use of critical services, like FP, in poorer urban contexts (8, 9).

Two articles in this issue present studies focusing on youthful populations in Conakry (Guinea). A qualitative study by Bangoura et al., drawing upon in-depth interviews and focus groups, provides insights into the experiences and decision-making processes of young people. Taking a similar approach, Dioubaté et al. examine the barriers to contraceptive use facing the youth. Collectively, these findings add to our existing knowledge of the wide range of dilemmas and competing influences that urban residents, especially young people, face in deciding whether or not to employ particular contraceptive methods. In contributing to this knowledge base, this work presents policymakers with specific factors that can be targeted to influence young people's preferences and motivations for FP uptake and use.

## The Way Forward

All the articles assembled in this Research Topic offer insights into under-researched places, groups and phenomena. However, as noted by Duminy et al., significant gaps in our knowledge remain. They argue that future research should prioritize themes such as neighborhoods and poverty, governance, migration and displacement, and resilience. Moreover, there is a need for work examining specific issues such as health systems responsiveness, innovative uses of technology, and cultural dynamism. Meeting these challenges demands an interdisciplinary approach that can inform a closer alignment between the FP and urban development sectors. This Research Topic of articles is one step in that direction.

## Author Contributions

JD led draft writing and revision. OO, MA, and MT reviewed and commented on drafts and approved final submission. All authors contributed to the article and approved the submitted version.

## Funding

This work was supported, in whole or in part, by the Bill & Melinda Gates Foundation (INV-008737) through the IUSSP Family Planning, Fertility and Urban Development programme. Further support was provided by the PEAK Urban programme, funded by UKRI's Global Challenges Research Fund, Grant Ref: ES/P011055/1.

## Conflict of Interest

OO was employed by Vital Strategies. The remaining authors declare that the research was conducted in the absence of any commercial or financial relationships that could be construed as a potential conflict of interest.

## Publisher's Note

All claims expressed in this article are solely those of the authors and do not necessarily represent those of their affiliated organizations, or those of the publisher, the editors and the reviewers. Any product that may be evaluated in this article, or claim that may be made by its manufacturer, is not guaranteed or endorsed by the publisher.
